# Spectrum of Visual Dysfunction Detected by a Novel Testing Protocol Within a Special School Eye Care Service

**DOI:** 10.22599/bioj.391

**Published:** 2024-11-11

**Authors:** Mohammud Musleh, Alison Green, Aleks Mankowska, Catherine Viner, Rachel Pilling

**Affiliations:** 1 York Hospital, UK; 2 University of Bradford, UK; 3 University of Bradford, Bradford Royal Infirmary, UK

**Keywords:** Cerebral visual impairment, Children’s visual acuity, Competing Interests

## Abstract

**Introduction:**

Children with special educational needs are more likely to have vision problems than peers in mainstream education. Reports focus on visual acuity and refraction, overlooking visuoperceptual difficulties, including cerebral visual impairment. This article reports on the feasibility and outcomes of visual function testing performed during in-school visual assessments.

**Method:**

A retrospective chart review was undertaken of children participating in a special school vision programme. The testing strategy included acuity, fields, contrast sensitivity, eye movements, accommodation, stereopsis, visual attention, refraction and a parent-completed questionnaire. The testing method was chosen based on the child’s ability to engage with testing.

**Results:**

78 cases were identified (mean age 9.6 years). Low vision (worse than 6/19) was identified in 31%. All six tests of visual function were completed by 44% (mean 5.1; range 2–6). The mean number of atypical responses was 1 (range 0–4). Almost half (49%) showed at least one atypical response, most commonly visual attention (35%) and 25% had atypical eye movements.

**Discussion:**

Overall, only 20% of children in the special school setting demonstrated a normal response to each test they were able to complete. Acuity testing alone does not demonstrate the atypical visual function of a child with special needs. Using techniques which require minimal cognitive, speech or motor function, a range of visual functions can be elicited during in-school testing. This testing strategy has the advantage of demonstrating areas of visual (dys)function impacting on the child’s ability to access learning which can be immediately fed back to teaching staff and carers.

## Introduction

There have been several reports examining the ocular and visual characteristics of children in special needs education in the past two decades (Black *et al.*, 2019; Das *et al.*, 2010; Donaldson *et al.*, 2019; Pilling and Outhwaite, 2017; Woodhouse *et al.*, 2014). It has been established that children with special educational or additional needs are more likely to have vision problems than peers in mainstream education, yet are less likely to have seen an optometrist (Donaldson *et al.*, 2019; Woodhouse, 1998). Reports tend to focus on acuity, refraction and fundus assessment. In recent years, the high prevalence of visual perceptual and processing difficulties, collectively known as cerebral visual impairment (CVI), amongst the special needs population has become evident (Chang and Borchert, 2020; Chokron and Dutton, 2023; Duke *et al.*, 2022).

The accepted definition of CVI is a verifiable visual dysfunction not attributable to deficits in the anterior visual pathways, or any co-existing ocular abnormality (Sakki *et al.*, 2018). CVI is understood to be the complex interaction of visual acuity, attention, field, motor and spatial perception and cognition. It has been established that abnormal visual perception adversely affects a child’s quality of life (Goodenough *et al.*, 2021).

A multidisciplinary approach to CVI diagnosis is necessary given the lack of formal diagnostic procedures and guidelines. There is no consensus about the nature or extent of the ophthalmological examination (Bauer *et al.*, 2022; Boonstra *et al.*, 2022; Cerebral Visual Impairment (CVI) Diagnostic Pathway – Visual Impairment Network for Children & Young People, 2015; Ortibus *et al.*, 2019; Pilling *et al.*, 2023). It is accepted such testing requires assessment of visual function beyond visual acuity and refraction.

Various diagnostic paradigms have been outlined with resources and settings ranging from detailed research environments to in-school testing (Philip *et al.*, 2016; Sakki *et al.*, 2021; Williams *et al.*, 2021). Whilst many stop short of formal visuoperceptual testing, all approaches include at minimum basic functional assessments of oculomotor, contrast sensitivity, visual field and visual acuity, in addition to fundoscopy, as part of a diagnostic framework (Galli *et al.*, 2022). Significant clinical heterogeneity is the key factor limiting the standardisation of testing and diagnosis of CVI. One study which sought to subtype CVI reported that 32% of subjects were unable to complete all tests of visual function (Sakki *et al.*, 2021). This was more evident in those with more severe visual and cognitive or motor impairment. In these cases, the authors determined a diagnosis of CVI based on experienced clinical judgement of visual function in the absence of formal visuoperceptual assessment.

A visual function (as opposed to visuoperceptual) approach to detecting CVI-related visual dysfunction is not novel. It is acknowledged that atypical visual function can be subjectively identified in children lacking cognitive, communication or motor skills by an experienced practitioner (Pehere and Jacob, 2019; Sakki *et al.*, 2021). There are several validated tools which have emerged that can supplement this approach using forced-choice preferential looking or reflex visual behavioural techniques (Bowman *et al.*, 2010; Elgohary *et al.*, 2017; Rossi *et al.*, 2017).

Parents and carers are well placed to report on visual difficulties which may not be evident during testing. The CVI5 questionnaire is a distillation of a 51-item inventory developed by Dutton and team (MacIntyre-Beon *et al.*, 2012). It is composed of five statements relating to activities of visual function. The respondent is asked to state if the child has difficulty with each are using a Likert scale. The questionnaire has been validated by other groups, who found the five questions had a sensitivity and specificity of 81% and 97%, respectively, for the diagnosis of CVI and recommended its use in large-scale screening studies to identify children for whom further CVI assessment is appropriate (Gorrie *et al.*, 2019).

The significance of CVI in children in special needs education has been acknowledged by the NHS England Special School Eye Care Service (SSECS) specification (NHSE SSECS Working Group Papers, unpublished). The SSECS recommended testing protocol includes an assessment of visual acuity, contrast, field, eye movements, accommodation, stereoacuity, visual attention and a parent-completed questionnaire to report symptoms of CVI (MacIntyre-Beon *et al.*, 2012). There is no guidance however as to which tests should be performed, or which children may warrant onward referral for CVI assessment.

The aim of this study is to report the feasibility and outcomes of including a specified set of reflexive visual function tests performed by an optometrist as part of a team conducting in-school visual assessment and examination which may support the identification of children with suspected CVI.

## Method

We undertook a retrospective review of the clinical records of all children who underwent assessment in two special school services in the north of England between January and August 2022, as part of an annual audit of the service. As part of ongoing quality improvement activities, ethical approval was not required.

All children who were not under the care of the hospital eye service were eligible for inclusion in the special school service programme. Parents were offered written information about the service and allowed to opt out. Table 1 shows the testing strategy. All tests were undertaken within the school environment, either the child’s classroom or a testing room within the school facility. Each child was accompanied by a teaching assistant who was familiar to them. To maximise engagement, there was no fixed testing protocol; rather tests were completed in an order determined individually by the practitioner. The practitioners (AM, CV, AG) worked as a team to elicit and record the clinical findings. All three practitioners have over 10 years experience of assessing children in a clinical setting and underwent the NHS England Special School Eye Care Service training and mentorship programme. The specific testing method was selected based on the child’s cognitive ability and attention, as determined by discussion with the teaching team and the practitioner’s observation of the child’s ability to engage adequately with each test. All tests were performed with both eyes open, wearing a refractive correction if this was available.

**Table 1:** Visual assessment strategy and outcomes. Visual acuity scores were categorised according to the World Health Organization, as adapted by Cumberland and Rahi (2016).


VISUAL FUNCTION	TEST CHOICE	NORMAL VALUES/OUTCOMES

Visual acuity	Bradford Visual Function Box (Pilling et al., 2016)	5 mm target or smaller – normal 8–50 mm target – low vision 70 mm target or larger – sight-impaired

	Cardiff Acuity Test Kay Picture Test (3 m, crowded) logMAR 3 m test (crowded)	6/6–6/12 – normal 6/15–6/48 – low vision 6/60 or worse – sight impaired

Visual field	Confrontation, using a target appropriate for child’s acuity threshold	Visual inattention in one or more hemifields, horizontal or vertical, or global suppression of peripheral field – atypical response

Contrast sensitivity	Hiding Heidi (Elgohary et al., 2017)	2.5% or better – normal 5% or worse – atypical response

Eye movements	Tracking at 30–50 cm (using a target appropriate for child’s acuity) Fast relocation movements between two objects at 30–50 cm, held approx. 30 cm apart	Smooth tracking – normal Delayed response, jerky or hesitant movement – atypical response Accurate movement – normal Inaccurate or inefficient movement – atypical response

Accommodation	Modified Bell Dynamic retinoscopy using an appropriately sized target at 30–40 cm (Tarczy-Hornoch, 2009)	Brisk change in reflex – normal Delayed or absent change in reflex – atypical response

Stereopsis	Lang Stereotest	Positive response to any test type – normal Uncertain or absence of response – atypical response

Visual Attention	Engagement with own reflection in a mirror moved progressively further away from child	<3 m – atypical response (based on age-matched norms and extrapolation to visual acuity) (Bowman et al., 2010)


As part of the NHS England protocol, prior to testing parents/carers were offered the opportunity to complete a questionnaire regarding the child’s previous eye care (‘About My Child’s Eyes’) which included the CVI5 questionnaire. A child reported to have difficulty ‘often’ or ‘always’ in three or more domains is considered to be significant.

## Results

The service undertook assessments on 78 children. The average age was 9.6 years (range 7–12 years). Overall, only 20% of children demonstrated a normal response to each test they were able to complete.

Visual acuity results are shown in Table 2. Visual acuity was recorded in all but one case (*n* = 77). The test chosen did not appear to be influenced by the age of the child, although there was a trend towards older children being able to engage with recognition, as opposed to preferential looking, tests: Bradford Visual Function Box (BVFB) (*n* = 17, average age 9.3), Cardiff Acuity Test (*n* = 36, average age 9.5), Kay Pictures (*n* = 15, average age 9.8) and LogMAR (*n* = 9, average age 10.5). Spectacles were worn by 15 patients during their assessment.

**Table 2:** Tools used to elicit visual acuity assessment. CC, Cardiff Acuity Test; BVFB, Bradford Visual Function Box. One patient could not engage with acuity measurement.


VISUAL ACUITY (*N* = 77)	TEST CHOICE	TEST UNDERTAKEN *N* (%)	OUTCOMES *N* (%)

	BVFB	17 (22%)	Normal 10 (59%) Low vision 7 (39%)

	CC	36 (47%)	Normal 18 (50%) Low vision 18 (50%)

	Kays	15 (19%)	Normal 11 (73%) Low 4 (27%)

	logMAR	9 (12%)	Normal 8 (89%) Low Vision 1 (11%)


Visual acuity was normal (6/15 or better) in 47 cases (61%) and low (6/19 or lower) in the remainder (*n* = 30, 38%). Six (7.7%) children were previously known to be registered sight impaired/severely sight impaired.

The results from the visual function testing are shown in Table 3. Just under half of patients (*n* = 34, 44%) were able to complete all six tests. The mean number of visual function domains a child was able to complete was 5.1 (range 2–6). The mean number of atypical responses was one (range 0–4). Thirty-eight (49%) of children had at least one atypical response, most commonly with visual attention (35%). One-quarter of children were found to have a deficit in eye movements or contrast sensitivity; one-fifth of children had atypical visual field responses. Accommodation was tested in all but one case. Stereopsis was formally tested in the fewest number of patients (*n* = 46, 59%).

**Table 3:** Results of visual assessment showing the proportion of children completing each domain of testing and proportion with a normal or atypical response.


VISION DOMAIN	NUMBER WHO UNDERTOOK TEST *N* (%)	OUTCOME *N* (% OF TOTAL UNDERTAKING TEST)

Visual field	71/78 (91%)	Normal 56 (79%) Atypical 15 (21%)

Contrast sensitivity	71/78 (91%)	Normal 53 (75%) Atypical 18 (25%)

Eye movements	70/78 (90%)	Normal 53 (76%) Atypical 17 (24%)

Accommodation	77/78 (99%)	Normal 76 (99%) Atypical 1 (1%)

Stereopsis	46/78 (59%)	Normal 43 (93%) Atypical 4 (9%)

Attention	63/78 (81%)	Normal 41 (65%) Atypical 22 (35%)

Parent-reported visual function (CVI5)	33 (42%)	Score 0–2 (normal) 29 (88%) Score 3–5 (atypical) 4 (12%)


Age did not appear to impact which children were able to complete all objective tests (mean 9.7 vs. 9.6 years). Considering the subset of children able to complete all tests, 65% (22) demonstrated normal visual function in all domains.

Six patients with low visual acuity completed all tests of visual function (30%). Stereopsis and accommodation were normal in all six, whereas contrast sensitivity was most often abnormal (4/6, 67%). Only one child (17%) with a low visual acuity showed typical responses on all six measures of wider visual function. A higher proportion of patients with normal visual acuity were able to complete all visual function tests (25/48, 52%). Here, all visual function tests were recorded as normal in the majority (19/25, 76%). Distance-maintained visual attention was most often abnormal (5/25, 20%).

The CVI5 questionnaire was completed by 33 (42%) families. Parents were more likely to complete this if their child had low vision (32% vs. 24%). There was no difference in the number of atypical test responses between the groups of children for whom a questionnaire had been returned compared with those with no questionnaire returned. The mean reported CVI5 score was 0.9 (range 0–5). Four families reported difficulties in three or more of the five questions (12%); The number of questions where ‘not applicable’ was selected ranged from 0 to 5 (average 1.8).

## Discussion

The results presented indicate a flexible testing protocol can facilitate the identification of visual functional deficits by eye health professionals in a special needs school setting. Whilst visual acuity may be normal in a high proportion of children in special needs education, only 20% of children have normal findings across all tests. When considering non-acuity visual function, almost half of children demonstrate atypical visual function in one or more domains. Research-based visuoperceptual testing batteries have been shown to be inaccessible by up to a third of those being assessed, most commonly those with complex needs (Sakki *et al.*, 2021). The approach described in this article has been shown to be suited to children with limited attention and engagement.

More than 20 years ago, the importance of proactively identifying visual problems in children at risk of neurodevelopmental problems was identified (Hård *et al.*, 2004). CVI-type visual dysfunctions based variously on assessment of field, eye movements, contrast, CVI5 and accommodation have been identified in cohorts of children with a third of children with Down syndrome (Wilton *et al.*, 2021), autistic spectrum disorder (Little, 2018) and one fifth- to one-half of children with cerebral palsy (Duke *et al.*, 2022). Amongst children with special needs, the cohort with complex needs has been reported to be highly likely to have moderate–severe visual impairment. One study reported that in over 32% of cases children were unable to complete the full assessment due to cognitive or motor impairment (Sakki *et al.*, 2021). In such cases, an experienced paediatric ophthalmologist uses observation of visual functioning, alongside excluding co-existing ocular causes for visual problems, to confirm the diagnosis. The availability of such clinicians to detect children in the community is limited. There is a need therefore to develop testing strategies which can identify those who may benefit from onward referral.

The impact of CVI on motor, social and cognitive development is sufficiently well-established to consider routine formal assessment of vision and visual perception in all children with neurodevelopment delays (Chokron and Dutton, 2023). However, the specific methods of such testing have not been defined outside of research contexts. This study proposes a potential screening model that has proven effective in real-life settings.

The use of questionnaires in identifying children with features suggestive of CVI is not new; however, our data suggest that given the high number of ‘not applicable’ responses, alternative questionnaires containing statements more applicable to a child with lower cognitive and/or motor function (Screening Tools | teach-cvi*), may warrant inclusion in future studies to better understand their role.

Our study has several strengths. The testing equipment is inexpensive, widely available in most eye services, portable, practicable to use in a range of environments and offers an inclusive approach to assessment. For each testing domain, a ‘reflexive’ testing option was available, that is the child does not require any instruction or direction to perform the test and therefore it is suitable for children with a broad range of acuities and cognitive abilities. This facilitated the assessors’ ability to demonstrate normal or atypical function. Our approach is in line with testing protocols including contrast, field, saccadic- and tracking-like eye movement which are being explored by several groups as a potential method of early identification of children at risk of developing CVI in infancy (Lee *et al.*, 2021; Rossi *et al.*, 2017).

The study is limited by the lack of available data on the children’s underlying medical conditions or co-existing diagnoses. The data may not be representative of all children with special needs, particularly those in a younger or older age group.

The testing protocol does not include visuoperceptual domains. Other studies have examined the role of visual crowding in identifying children with CVI-related visual dysfunction (van der Zee *et al.*, 2017; Williams *et al.*, 2021) and this metric would be useful for those children able to engage with optotype acuity assessment. Consideration of other methodologies to assess visual attention and eye movements such as fixation delay time would offer more objective reporting. Whilst it is acknowledged that children in special needs schools are more likely to have vision problems than those in mainstream schools, there is yet to be a consensus on which children should be offered onward referral for assessment for CVI. The present study lacks the ability to further refine a threshold for how many atypical visual domains would trigger a CVI assessment; however, we have presented the mechanism by which a larger study could determine the answer to this important question.

We have presented data from a heterogenous cohort of children from special schools to demonstrate which tests offer a high engagement rate. This represents the real-life testability of children in their own environment. This testing strategy has the advantage of demonstrating areas of visual (dys)function which are impacting on the child’s ability to access learning which can be immediately fed back to the teaching staff and carers. Whether or not the child is subsequently diagnosed with CVI, highlighting adaptations which may be made to support the child’s vision (e.g., area of best visual attention, ability to see moving objects, size and contrast of objects easily seen) is an important step in the child’s development.
